# DNMT1 genetic polymorphisms affect breast cancer risk in the central European Caucasian population

**DOI:** 10.1186/1868-7083-5-7

**Published:** 2013-05-02

**Authors:** Kathrin Kullmann, Mustafa Deryal, Mei Fang Ong, Werner Schmidt, Ulrich Mahlknecht

**Affiliations:** 1 Department of Internal Medicine, Division of Immunotherapy and Gene Therapy, José Carreras Research Center, Saarland University Medical Center, Homburg/Saar D-66421, Germany; 2 Department of Obstetrics and Gynecology, Saarland University Medical Center, Homburg/Saar D-66421, Germany; 3 Institute of Medical Biometrics, Epidemiology and Medical Informatics, Saarland University, Homburg/Saar D-66421, Germany; 4 Department of Hematology/Oncology, St Lukas Clinic Solingen, Schwanenstrasse 132, Solingen D-42697, Germany

**Keywords:** DNMT, SNP, Breast cancer

## Abstract

**Introduction:**

DNA methylation of CpG islands within the promoter region of genes is an epigenetic modification with an important role in the development of cancer and it is typically mediated by DNA methyltransferases (DNMTs). In cancer cells, global hypomethylation of the genome as a whole and regional hypermethylation of CpG islands have been reported. Four groups of DNMTs have been identified: DNMT1, DNMT2 (TRDMT1), DNMT3A and DNMT3B. DNMT2 uses the catalytic mechanism of DNMTs, but does in fact methylate RNA. Little is known about the significance of these genes in human breast cancer. In the study presented herein, we analyzed five distinct DNMT single SNPs with regard to potential associations with breast cancer risk.

**Case description:**

In this study, we genotyped 221 female Caucasian breast cancer patients and 221 female Caucasian healthy controls, and we used five allele-specific real-time polymerase chain reaction (qPCR) assays. We selected one locus within the *DNMT1* gene and two loci within the *DNMT3A* and *DNMT3B* genes, respectively. Statistics were calculated using the chi-squared and Fisher’s exact tests, and correlated with clinical parameters such as age, diagnosis, histology, TNM stage, hormonal receptor status, human epidermal growth factor receptor 2 (HER2) status, response to treatment and survival. Statistically significant results were obtained for correlations with the *DNMT1* gene.

**Discussion and Evaluation:**

Five genomic loci within the *DNMT1*, *DNMT3A* and *DNMT3B* genes were assessed. Statistical significance (*P* = 0.030) was identified for *DNMT1* SNP (A201G, rs2228612): six women within the control group were GG homozygous (variant), while this mutation was absent in the breast cancer group.

**Conclusions:**

We conclude that women with the *DNMT1* SNP (A201G, rs2228612) GG homozygous genotype (variant) have a lower risk of developing breast cancer compared to heterozygous or wildtype genotypes. To date, alterations within the *DNMT1* gene have not been reported to be associated with cancer in the Caucasian population.

## Background

Breast cancer is the most frequent malignancy in women and it is also the leading cause of death among women aged 40 to 50 years. Numerous risk factors of breast cancer have been reported in the literature and the most relevant factors are geographical variations, lifestyle, age at the time of diagnosis, age at first pregnancy, age at menarche, age at menopause and family history. *BRCA1* and *BRCA2* are genes associated with an increased risk of developing breast cancer. Inherited mutations in the *p53* and *PTEN* genes have been observed in the context of familial syndromes, Li-Fraumeni syndrome and Cowden’s disease, which imply an increased risk of developing breast cancer. However, we need to consider that both Li-Fraumeni syndrome and Cowden’s disease are rare conditions [1]. Epigenetic events are important in the pathogenesis and progression of breast cancer [2]. Histone acetylation and/or methylation, as well as DNA methylation, are epigenetic alterations which are reversible. Histone modifications take place in eukaryotic cells, while DNA methylation takes place in both eukaryotic and prokaryotic cells. Both are relevant key elements within the transcriptional regulatory machinery [3]. The epigenetic modification of DNA methylation is typically mediated by DNA methyltransferases (DNMTs). In cancer cells, there is a variation in 5-methylcytosine (m^5^C) transmission along with global DNA hypomethylation [4]. However, promoter CpG islands are typically hypermethylated in many types of cancers, which can lead to transcriptional silencing of the corresponding genes [5,6]. Accordingly, the hypermethylation of gene promoters or a hypomethylation of various parts of the genome can contribute to the development of cellular malignancy or autoimmune disease [7].

DNA methylation plays an important role in the control of gene expression in mammalian cells [8]. In breast cancer, it has been shown that the methylation of promoter regions in tumor suppressor genes can provide a growth advantage to malignant cells. For example the hypermethylation of the CpG islands in the estrogen receptor (ER)-promoter leads to the loss of ER protein expression. Therefore, the tumor is no longer under estrogen control and this causes the growth of cancer [9]. Nevertheless, with regard to human breast cancer, little is known about the clinical and biological relevance of DNMTs.

**Figure 1:**
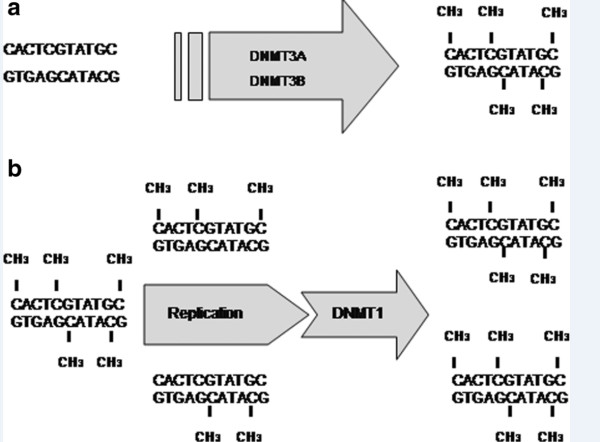
**The DNA methyltransferases (DNMTs) are divided into two groups on the basis of their functional activities: *de novo *(a) and maintenance of DNA methylation (b).***De novo* DNMTs (DNMT3A and DNMT3B) can create m^5^CpG dinucleotides from unmethylated DNA, while the maintenance of methyltransferase DNMT1 attaches methyl groups to hemimethylated DNA during replication.

To date, four mammalian DNMTs have been identified: DNMT1, DNMT2 (TRDMT1), DNMT3A and DNMT3B [10-12]. DNMT1 was the first methyltransferase to be found [13], and it is also the major and most well characterized DNMT [14]. DNMTs can be divided into two groups, which are responsible for *de novo* and the maintenance of methylation (Figure 1) [15]. DNMT1 associates with the DNA replication fork and binds methyl groups to hemimethylated DNA during replication for the maintenance of methylation in the genome. The expression of DNMT1 is regulated by microRNAs (in breast cancer tissues microRNAs are globally downregulated) [16] and the methyltransferase activity is reduced by phosphorylation of DNMT1 [17]. Both mechanisms are involved in the regulation of global DNA methylation, but phosphorylation has been directly associated with tumorigenesis [17]. However, the precise role of DNMT1 functions in cancer cells is less well understood, since alterations of this gene have not been reported for cancer [18].

DNMT3A and DNMT3B are mainly involved in *de novo* methylation and they are important for the generation of methylation patterns during embryogenesis [19]. *De novo* DNMTs methylate cytosine to m^5^C post-replicatively from unmethylated DNA [3]. DNMT3A is also able to methylate cytosine within CpA and CpT dinucleotides, although this enzyme is highly specific for CpG methylation. However, the function of this DNA modification is still unknown [20]. The two functionally different groups of DNMTs may also interact with each other and activate HDAC1, a histone deacetylase, which represses gene expression through the deacetylation of histone proteins, and thus a conformational change of the chromatin architecture [3]. DNMT2 (TRDMT1) is not only found in humans, but it is also found in other mammalian and non-mammalian species, and exhibits functional activities that are distinct from the other DNMTs. DNMT2 is an active RNA methyltransferase which is responsible for the methylation of the tRNA^Asp^ cytosine in position 38 (C38) [21]. It is the first example of an RNA methyltransferase to take advantage of the catalytic mechanisms of DNMT [22].

In order to assess the relevance of *DNMT1*, *DNMT3A* and *DNMT3B* SNPs on *DNMT* gene expression and associated enzymatic activities, a number of gene loci were identified *in silico* on the basis of the information obtained from literature and SNP databases: *DNMT1* SNP (A201G, rs2228612), *DNMT3A* SNPs (G301C, rs34843713 and G301A, rs34191084) and *DNMT3B* SNPs (C501T, rs406193 and G301A, rs35846833). In this context, the *DNMT2* (*TRDMT1*) gene has not been considered, because human DNMT2 is an RNA methyltransferase and this study was primarily focused on genes coding for DNMTs, and because they have been reported to play an important role in the development of cancer [3].

With the study presented herein, it was our intention to assess whether there was a correlation between *DNMT* SNPs and the risk of developing breast cancer.

## Case description

### Blood and peripheral blood mononuclear cell (PBMC) samples

In this study, we analyzed 221 DNA samples isolated from PBMCs of female Caucasian breast cancer patients and 221 DNA samples from female Caucasian healthy donors. Patient samples were collected at the Department of Obstetrics and Gynecology, Saarland University Medical Center, Germany, between 2001 and 2010. Control samples from healthy donors were collected at the Central Laboratory, University of Heidelberg Medical Center, Germany. All patients in the control group were below the age of 30 years. Genomic DNA was isolated from PBMCs and whole blood samples using the NucleoSpin Blood kit (Macherey-Nagel, Düren, Germany), in accordance with the manufacturer’s instructions. Analyses of DNA purity and quantification were performed using a NanoDrop spectrophotometer (Thermo Scientific, Waltham, MA, USA).

### SNP selection

We identified SNPs within the coding and promoter regions of the *DNMT1*, *DNMT3A* and *DNMT3B* genes via *in silico* analyses using data from the National Center for Biotechnology Information (NCBI, Bethesda, MD, USA), Applied Biosystems (Life Technologies, Carlsbad, CA, USA), GeneCards and HapMap. These SNPs carried the potential to affect the qualitative and quantitative expression of the DNMTs mentioned, and to search for potential associations with clinical parameters of breast cancer patients (Additional file 1 and Table 1). Five genomic loci corresponded to the criteria mentioned, and were further analyzed in the group of female Caucasian breast cancer patients and controls: *DNMT1* SNP (A201G, rs2228612), *DNMT3A* SNPs (G301C, rs34843713 and G301A, rs34191084) and *DNMT3B* SNPs (C501T, rs406193 and G301A, rs35846833). *DNMT3B* SNP (C501T, rs406193) was selected on the basis of the publication by Cebrian *et al*. [2], which showed a reduced risk of breast cancer for the T allele (variant) within the *DNMT3B* gene. The *DNMT1* SNP (A201G, rs2228612) was recently analyzed in Chinese women by Sun *et al*. [23] and they were able to demonstrate breast cancer susceptibility in women with the GG homozygote genotype (variant) [23]. The other SNPs were selected on the basis of non-synonymous SNPs, which could impact gene expression and/or protein function, because they lead to a modified amino acid product.

**Table 1:** Distribution of the clinical parameters

**Clinical parameters**		**Distribution (%)**
**Age (years)**	20 to 40	2.30
	41 to 60	31.70
	61 to >80	66.10
**Breast cancer diagnosis**	Right breast	47.10
	Left breast	46.10
	Both	6.80
**Histology**	Ductal	67.40
	Lobular	15.40
	DCIS	6.40
	LCIS	1.80
	Mixed and other tumor types	9.00
**TNM stage**	I	36.70
	IIa	24.40
	IIb	10.90
	IIIa	8.60
	IIIb	5.00
	IIIc	8.10
	IV	6.30
**ER status**	Positive	82.40
	Negative	17.60
**PR status**	Positive	72.40
	Negative	27.60
**HER2 status**	Positive	14.00
	Negative	67.00
	Not defined	19.00
**Response of chemotherapy**	No chemotherapy/data	48.00
	Complete remission	32.10
	Recurrence	5.90
	Stable disease	8.10
	Progress	5.90
**Survival**	Not detected	10.90
	Alive	76.00
	Death	13.10

DCIS, ductal carcinoma *in situ*; ER, estrogen receptor; HER2, human epidermal growth factor receptor 2; LCIS, lobular carcinoma *in situ*; PR, progesterone receptor; TNM, TNM Classification of Malignant Tumours.

### Genotyping

All samples of the selected tag SNPs were genotyped by real-time polymerase chain reaction (qPCR) or on TaqMan probes (Applied Biosystems, Darmstadt, Germany). qPCRs were carried out on 10 ng of genomic DNA using TaqMan universal PCR Master Mix (Applied Biosystems, Darmstadt, Germany). Forward and reverse primers were labeled VIC and FAM (designed by Applied Biosystems) in a 5 μl reaction (Table 2). The amplification conditions of TaqMan were as follows: 5 seconds at 50°C, followed by 10 minutes at 95°C and 12 seconds at 92°C, and finally 1 minute at 60°C. A total of 40 cycles were run and the completed PCRs were then read in the end-point mode using the Allelic Discrimination Sequence Detector Software (Applied Biosystems). A total of 221 DNA samples from female Caucasian breast cancer patients and 221 DNA samples from female Caucasian healthy controls were assessed (Table 3).

**Table 2:** Oligonucleotide primers

**rs number**	**Gene**	**SNP**	**AA**	**Position**	**Chromosome**	**Target sequence**	**Codon**
**rs2228612**	***DNMT1***	**A201G**	**I/V**	**Exon**	**19**	**5′-CAGAAA(C/T)CTGTGG-3′**	**327**
**rs34843713**	***DNMT3A***	**G301C**	**R/P**	**Exon**	**2**	**5**′**-AAGGGG(C/G)GATCAT-3**′	**749**
**rs34191084**	***DNMT3A***	**G301A**	**G/S**	**Exon**	**2**	**5**′**-CATCGC(C/T)TGCTTT-3**′	**278**
**rs406193**	***DNMT3B***	**C501T**	**-**	**Intron**	**20**	**5**′**-GAGACC(C/T)ATTAAT-3**′	**-**
**rs35846833**	***DNMT3B***	**G301A**	**R/C**	**Exon**	**20**	**5**′**-CGAAGA(C/T)GCACAG-3**′	**320**

**Table 3:** Genotype distribution as listed in the NCBI database compared to data obtained in this study

**SNP**	**Number of samples (NCBI data)**	**Number of samples (study results)**	**Wildtype (NCBI data)**	**Wildtype (study results)**	**Heterozygote (NCBI data)**	**Heterozygote (study results)**	**Variant (NCBI data)**	**Variant (study results)**
**DNMT1 SNP**	226	221^a^	88.50%	87.3%^a^	11.50%	12.7%^a^	5.80%	0%^a^
(A201G, rs2228612)		221^b^		81.4%^b^		15.8%^b^		2.7%^b^
**DNMT3A SNP**	74	221^a^	97.30%	100%^a^	2.70%	0%^a^	1.40%	0%^a^
(G301C, rs34843713)		221^b^		100%^b^		0%^b^		0%^b^
**DNMT3A SNP**	68	221^a^	97.10%	100%^a^	2.90%	0%^a^	1.50%	0%^a^
(G301A, rs34191084)		221^b^		100%^b^		0%^b^		0%^b^
**DNMT3B SNP**	120	221^a^	75%	69.7%^a^	25%	28%^a^	12.50%	2.3%^a^
(C501T, rs406193)		221^b^		77.8%^b^		20.8%^b^		1.4%^b^
**DNMT3B SNP**	74	221^a^	97.30%	100%^a^	2.70%	0%^a^	1.40%	0%^a^
(G301A, rs35846833)		221^b^		100%^b^		0%^b^		0%^b^

^a^Results of cases; ^b^results of controls. NCBI, National Center for Biotechnology Information.

### Statistical methods

The Hardy-Weinberg equilibrium was tested using the Simple Hardy-Weinberg Calculator by Michael H Court ( http://www.tufts.edu/~mcourt01/Documents/Court%20lab%20-%20HW%20calculator.xls), by comparing the observed and expected genotype frequencies for cases and controls. Genotyping data generated for patients and controls were correlated with clinical parameters (age, diagnosis, histology, TNM status, ER status, progesterone receptor (PR) status, human epidermal growth factor receptor 2 (HER2) status, response to chemotherapy and survival) on the basis of the chi-squared and Fisher’s exact tests. P values ≤0.05 were considered to be statistically significant. All analyses were performed using the statistical analysis software SPSS Statistics Version 17 for Windows (IBM, Armonk, NY, USA).

Body which gave approval: Ärztekammer des Saarlandes, Körperschaft des öffentlichen Rechts, Ethik-Kommission. Reference number: 192/09.

## Evaluation

The correlation of the genotyping results with clinical parameters (age at the time of diagnosis, duration of disease, histology, TNM status, ER status, PR status, HER2 status, response of chemotherapy and survival) revealed no statistical significance. The correlation of the genotyping results between cases and controls showed no coherence for the *DNMT3A* SNPs (G301C, rs34843713 and G301A, rs34191084) and *DNMT3B* SNP (G301A, rs35846833). All women were homozygous for the *DNMT3A* GG and *DNMT3B* GG genotype. Based on information obtained from the NCBI database, the frequency of this polymorphism within the Caucasian population was reported to be 1.4% for the *DNMT3A* SNP (G301C, rs34843713) on the basis of 74 samples, 1.4% for the *DNMT3B* SNP (G301A, rs35846833) on the basis of 74 samples and 1.5% for the *DNMT3A* SNP (G301A, rs34191084) on the basis of 68 samples (Table 3).

The *DNMT3B* SNP (C501T, rs406193) was analyzed earlier as reported in the literature [2]. While Cebrian et al. showed that there was a significant difference in genotype distribution between breast cancer patients and controls, with the T allele (variant) associated with a reduced risk for breast cancer [2], our own analyses did not show a statistically significant correlation between the *DNMT3B* SNP and corresponding clinical parameters. Our genotyping data comparing patients and controls varied between 1.4% and 2.3% (Table 3), and were clearly lower than the frequencies reported in the NCBI database (12.5% for *DNMT3B* SNP (C501T, rs406193) in central European Caucasian females) (Table 3). This analysis identified a statistical significance with a P value of 0.05.

We identified one additional statistically significant correlation for *DNMT1* SNP (A201G, rs2228612), which appears to protect women against developing breast cancer.

**Figure 2:**
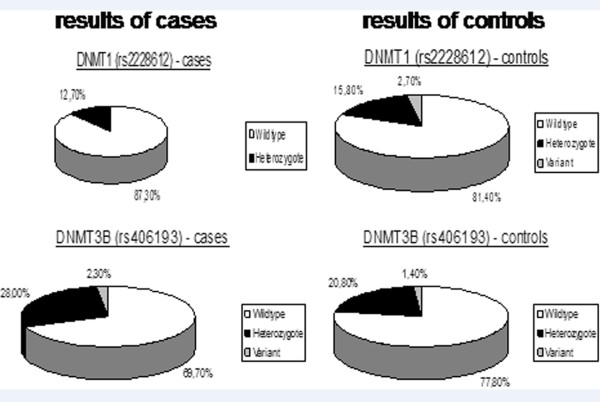
**Genotyping results.** In this study, we genotyped 221 female Caucasian breast cancer patients and 221 female Caucasian healthy controls. There was a statistical significance in the DNMT1 gene (*DNMT1* SNP (A201G, rs2228612)) between cases and controls (*P* = 0.03). The genotyping results in the *DNMT3B* gene (DNMT3B SNP (C501T, rs406193)) between cases and controls were also statistically significant (*P* = 0.05).

The *P* value of our results was 0.030, in accordance with Fisher’s exact test. The genotyping results showed that within the control group, 6 out of 221 women were GG homozygous (variant), while the homozygous variant genotype was not found in the group of breast cancer patients (Figure 2 and Table 3). Therefore, our results indicate that the G allele (variant) is associated with a reduced risk of developing breast cancer.

## Discussion

In the case–control study presented herein, we correlated genetic polymorphisms in three genes, *DNMT1*, *DNMT3A* and *DNMT3B*, with clinical parameters to consider the risk of female Caucasians developing breast cancer.

The correlation with the SNPs in the *DNMT* genes and the clinical parameters showed no statistical significance. The identification of SNP alterations in association with other risk factors for breast cancer could provide an awareness of genetic variants that could lead to an increased susceptibility of breast cancer [24]. Milne et al. failed to identify conclusive associations between the 12 selected SNPs and the age at menarche, parity, age at first birth or body mass index (BMI) [24]. However, none of the *DNMT* SNPs tested in our study were considered in the study by Milne et al. [24]. Therefore, correlation of *DNMT* SNPs with clinical parameters and/or environmental risk factors such as breastfeeding, height, oral contraceptive and menopausal hormone therapy use, alcohol consumption, cigarette smoking and physical activity, could potentially yield significant insights of breast cancer risk, which may then be considered for the design of further studies in the future [25].

Two out of five SNPs in the *DNMT1*, *DNMT3A* and *DNMT3B* genes (*DNMT3B* SNP (C501T, rs406193) and *DNMT1* SNP (A201G, rs2228612)) reached statistical significance (P = 0.05 and 0.03, respectively). For DNMT3A, we assessed two SNPs (G301C, rs34843713 and G301A, rs34191084), which revealed the homozygous wildtype allele in all samples that were studied (cases and controls). *DNMT3A* SNPs with an impact on protein levels would have been of special interest, since Ley et *a*l. showed that DNMT3A mutations are highly recurrent in patients with *de novo* acute myelogenous leukemia (AML) and with an intermediate-risk cytogenetic profile, and these patients also had a significantly reduced survival [26]. Furthermore, there is also a coherence between DNMT3A mutations and patients with myelodysplastic syndrome (MDS), since Walter et al. described the frequency of DNMT3A mutations in patients with *de novo* MDS and their association with secondary AML [27]. These mutations correlated with overall reduced survival and accelerated progression to AML [27]. AML and chronic myelogenous leukemia (CML) cells in the acute phase also showed increased expression levels for DNMT1, DNMT3A and DNMT3B when compared to normal bone marrow cells. However, such an over-expression of methyltransferases was not observed in the chronic phase in CML cells [28]. Since DNMT3B is able to mediate *de novo* DNA methylation and has been shown to be over-expressed in numerous types of cancer [14,28-31], this methyltransferase has been postulated to be an important factor in cancer, and it has been shown that DNMT3B polymorphisms are in fact significantly associated with the risk of developing lung cancer [32,33]. The level of DNMT3B protein is significantly elevated in hypermethylated human breast cancer cell lines, leading to an increased DNMT activity and high rates of methylation-dependent gene silencing [31]. This is in accordance with other studies, which revealed an association of over-expressed DNMT3B levels and the development of breast cancer [8] and other malignancies [14,28-31].

In this study, we assessed two interesting *DNMT3B* SNPs, of which *DNMT3B* SNP (G301A, rs35846833) failed to be confirmed in the female Caucasian population, showing only the *wildtype* genotype in all samples. *DNMT3B* SNP (C501T, rs406193) showed a significant difference among cases and controls (P value = 0.05) with the T allele (variant) associated with a reduced risk of developing breast cancer, while the statistical analysis of the correlation with clinical parameters was not significant. Montgomery et al. described a strong association between the C allele of the DNMT3B promoter polymorphism (C-149 T, rs2424913) and the risk of developing breast cancer, compared to TT homozygotes [34].

Ye et al. genotyped a total of 47 SNPs in the *DNMT1* and *DNMT3B* genes, and the *DNMT3B* SNP (C501T, rs406193), which was also analyzed in our study [35]. Twenty-two of these SNPs, including the *DNMT3B* SNP (C501T, rs406193), were rejected from the analysis because either the minor allele frequencies were less than 0.005 in the study population or they were found to deviate from the Hardy-Weinberg equilibrium in controls. Thirteen SNPs in the *DNMT3B* gene (rs6058869, rs242908, rs6119954, rs6141813, rs4911108, rs4911259, rs910084, rs6088008, rs998382, rs4911110, rs6058893, rs6058896, rs8118663) were included in the analysis, but Ye et al. observed no association between these SNPs and breast cancer among Chinese women [35], which was inconsistent with the result of Cebrian et al. [2]. Cebrian et al. [2] showed statistical significance for *DNMT3B SNP* (C501T, rs406193) (odds ratio (OR) CT versus CC = 0.85 (0.77 to 0.94); OR TT versus CC = 0.89 (0.64 to 1.23); P heterogeneity = 0.007; *P* trend = 0.003). This association was in accordance with our statistical analysis, but Ye et al. could not confirm an apparent association of the DNMT3B polymorphism (C501T, rs406193) with the risk of breast cancer among Chinese women, since this SNP was found to have a minor allele frequency of less than 0.05 in the study population and was excluded from the analysis [35]. Consequently, we suggest that the mutation in the *DNMT3B* gene (C501T, rs406193) is only relevant in the female Caucasian population and not among the female Chinese population, which might be due to the difference of the allele frequency of this mutation between Caucasian (12.5%) and Chinese patients (1.2%), according to the NCBI database (since genetic mutations often vary between ethnic groups).

DNMT1 is a maintenance methyltransferase which attaches methyl groups to hemimethylated DNA during replication. To date, no genetic polymorphisms have been reported for DNMT1 in association with breast cancer in the female Caucasian population [18]. In the study presented herein, we identified one *DNMT1* SNP (A201G, rs2228612) which could be relevant as a risk of developing breast cancer in the female Caucasian population. In relation to breast cancer risk, *DNMT1* gene polymorphisms have been solely reported in association with sporadic infiltrating ductal breast cancer among Chinese women [36]. Xiang et al. studied two SNPs in the *DNMT1* gene (T251C, rs16999593 and G301A, rs2228611), and reported significance between the SNPs and the PR and p53 status (p53-positive disease) [36]. Ye et al. analyzed 12 SNPs in the *DNMT1* gene (rs2116940, rs2336691, rs7253062, rs16999593, rs6511685, rs6511677, rs8101866, rs2241531, rs10418707, rs10407514, rs4804122, rs11085587), but they did not find any apparent association of DNMT1 mutation with the risk of breast cancer among Chinese women [35]. A recent article by Sun et al. found evidence of an association of breast cancer risk among Chinese Han women from South China with SNPs in the *DNMT1* gene [23]. For *DNMT1* SNP rs2228612, the frequency of the GG genotype (variant) of rs2228612 was higher in cases compared to controls (22.5% versus 14.5%) (OR AG versus GG = 1.71 (1.06 to 2.78); OR GG versus AA 1.75 (1.13 to 2.72); *P* heterogeneity = 0.044; *P* trend = 0.013) [23]. These results contradict our own observations, which imply ethnic differences still remain to be further elucidated.

In this study, we were able to show a significant association between the *DNMT1* gene polymorphism (A201G, rs2228612) and the risk of breast cancer in the female Caucasian population. The correlation of cases and controls showed a statistical significance with a P value of 0.030 (Fisher’s exact test), whereas the statistical analyses of frequency differences between cases and clinical parameters revealed no significant results. While six female controls were GG homozygous (variant), there were no females in the group of breast cancer patients with the GG homozygous genotype (variant). Therefore, our results show that the *DNMT1* gene polymorphism (A201G, rs2228612) with the g-allele (variant) is associated with a reduced risk of developing breast cancer.

## Conclusion

In conclusion, our results showed that the two *DNMT3A* SNPs (G301C, rs34843713 and G301A, rs34191084) and the *DNMT3B* SNP (G301A, rs35846833) do not exist in the female Caucasian population. Otherwise, we could demonstrate a statistical significance in the differences of allele frequencies of cases and controls of the *DNMT3B* SNP (C501T, rs406193), like in the study of Cebrian et al. [2].

Finally, we identified a significant association of the *DNMT1* SNP (A201G, rs2228612) variant in the correlation of cases and controls. Women with the G allele (variant) show a lower risk of developing breast cancer. The diagnostic impact of this new marker needs to be validated in further clinical studies in larger populations and within different ethnic groups, but it presents a further step towards a more individualized diagnosis.

## Abbreviations

AML: Acute myelogenous leukemia; BMI: Body mass index; C: Cytosine; CML: Chronic myelogenous leukemia; DNMT: DNA methyltransferase; ER: Estrogen receptor; HER2: Human epidermal growth factor receptor 2; m5C: 5-methylcytosine; MDS: Myelodysplastic syndrome; NCBI: National Center for Biotechnology Information; OR: Odds ratio; PBMC: Peripheral blood mononuclear cell; PR: Progesterone receptor; qPCR: Real-time polymerase chain reaction; SNP: Single nucleotide polymorphism; TNM: TNM Classification of Malignant Tumours.

## Competing interest

All authors declare that they have no conflict of interest in association with this study.

## Authors’ contributions

KK carried out the molecular genetic studies and performed the statistical analysis with MFO. UM planned the study. KK and UM worked on the manuscript. All authors read and approved the final manuscript.

## Supplementary Material

Additional file 1: Table S1 Data of breast cancer patients. **Table S2.** Explanation of the clinical parameters. The age of the patients are defined as age at the time of recruitment. Click here for file
