# Supplementary material for: DNMT1 genetic polymorphisms affect breast cancer risk in the central European Caucasian population
Source: Clin Epigenetics. 2013 May 2;5(1):7. doi: 10.1186/1868-7083-5-7 (PMC3646668; doi:10.1186/1868-7083-5-7)
Supplement: Additional file 1: Table S1 — Data of breast cancer patients. Table S2. Explanation of the clinical parameters. The age of the patients are defined as age at the time of recruitment. [file 1868-7083-5-7-S1.doc]

| **patient no.** | **age** | **diagnosis** | **histology** | **TNM stadium** | **ER status** | **PR status** | **Her2 status** | **response of chemotherapy** | **survival** |
| --- | --- | --- | --- | --- | --- | --- | --- | --- | --- |
| **1** | **3** | **1** | **5** | **2** | **0** | **0** | **0** | **0** | **1** |
| **2** | **2** | **3** | **1** | **7** | **1** | **1** | **0** | **1** | **1** |
| **3** | **3** | **2** | **1** | **7** | **1** | **1** | **0** | **1** | **1** |
| **4** | **2** | **1** | **2** | **7** | **0** | **0** | **1** | **4** | **1** |
| **5** | **3** | **1** | **1** | **1** | **0** | **1** | **0** | **1** | **1** |
| **6** | **3** | **1** | **2** | **6** | **1** | **1** | **0** | **4** | **1** |
| **7** | **3** | **2** | **1** | **3** | **1** | **0** | **0** | **1** | **2** |
| **8** | **2** | **1** | **1** | **3** | **1** | **1** | **0** | **1** | **0** |
| **9** | **3** | **2** | **1** | **4** | **1** | **0** | **0** | **1** | **0** |
| **10** | **3** | **2** | **5** | **2** | **0** | **0** | **0** | **1** | **0** |
| **11** | **3** | **2** | **3** | **1** | **0** | **0** | **0** | **1** | **1** |
| **12** | **3** | **2** | **2** | **3** | **1** | **0** | **0** | **0** | **0** |
| **13** | **3** | **1** | **1** | **7** | **1** | **1** | **1** | **4** | **2** |
| **14** | **3** | **1** | **1** | **1** | **1** | **1** | **0** | **1** | **1** |
| **15** | **3** | **1** | **1** | **1** | **0** | **1** | **0** | **1** | **1** |
| **16** | **2** | **2** | **3** | **1** | **1** | **1** | **1** | **1** | **1** |
| **17** | **3** | **1** | **1** | **1** | **1** | **1** | **1** | **4** | **1** |
| **18** | **3** | **2** | **2** | **5** | **1** | **1** | **1** | **1** | **0** |
| **19** | **3** | **2** | **2** | **3** | **1** | **1** | **0** | **1** | **1** |
| **20** | **3** | **1** | **2** | **1** | **1** | **1** | **0** | **0** | **1** |
| **21** | **3** | **2** | **1** | **4** | **1** | **0** | **0** | **0** | **2** |
| **22** | **3** | **2** | **1** | **2** | **1** | **0** | **0** | **1** | **1** |
| **23** | **3** | **2** | **1** | **3** | **1** | **1** | **0** | **1** | **1** |
| **24** | **2** | **1** | **4** | **1** | **1** | **1** | **0** | **2** | **0** |
| **25** | **1** | **2** | **1** | **1** | **0** | **0** | **0** | **1** | **1** |
| **26** | **2** | **1** | **1** | **2** | **1** | **0** | **0** | **1** | **1** |
| **27** | **3** | **2** | **1** | **3** | **1** | **1** | **0** | **1** | **1** |
| **28** | **3** | **1** | **2** | **7** | **1** | **0** | **0** | **1** | **1** |
| **29** | **3** | **2** | **2** | **1** | **1** | **1** | **0** | **1** | **1** |
| **30** | **3** | **2** | **2** | **1** | **1** | **0** | **0** | **1** | **1** |
| **31** | **3** | **2** | **1** | **2** | **1** | **1** | **0** | **1** | **1** |
| **32** | **2** | **3** | **1** | **2** | **0** | **1** | **0** | **1** | **1** |
| **33** | **3** | **1** | **1** | **2** | **1** | **1** | **0** | **1** | **1** |
| **34** | **3** | **3** | **4** | **7** | **1** | **0** | **0** | **1** | **1** |
| **35** | **3** | **2** | **2** | **7** | **1** | **1** | **0** | **2** | **1** |
| **36** | **3** | **1** | **1** | **1** | **1** | **1** | **0** | **1** | **1** |
| **37** | **3** | **2** | **2** | **1** | **1** | **1** | **2** | **0** | **1** |
| **38** | **3** | **1** | **1** | **1** | **1** | **0** | **0** | **1** | **1** |
| **39** | **3** | **1** | **5** | **1** | **1** | **1** | **0** | **0** | **2** |
| **40** | **3** | **1** | **5** | **1** | **1** | **0** | **0** | **1** | **1** |
| **41** | **3** | **1** | **2** | **6** | **1** | **1** | **0** | **1** | **1** |
| **42** | **3** | **1** | **1** | **1** | **1** | **0** | **0** | **1** | **1** |
| **43** | **3** | **1** | **1** | **3** | **1** | **0** | **1** | **1** | **1** |
| **44** | **3** | **1** | **1** | **2** | **1** | **0** | **0** | **1** | **1** |
| **45** | **3** | **2** | **1** | **4** | **1** | **0** | **1** | **0** | **1** |
| **46** | **3** | **2** | **1** | **3** | **1** | **0** | **0** | **1** | **1** |
| **47** | **2** | **1** | **1** | **1** | **0** | **0** | **0** | **1** | **1** |
| **48** | **3** | **1** | **1** | **2** | **1** | **1** | **0** | **4** | **2** |
| **49** | **3** | **1** | **1** | **2** | **1** | **0** | **0** | **0** | **1** |
| **50** | **3** | **1** | **1** | **1** | **1** | **1** | **0** | **0** | **1** |
| **51** | **3** | **1** | **1** | **1** | **1** | **1** | **0** | **0** | **1** |
| **52** | **3** | **2** | **1** | **2** | **0** | **1** | **0** | **1** | **1** |
| **53** | **3** | **2** | **1** | **5** | **0** | **0** | **1** | **0** | **1** |
| **54** | **3** | **2** | **2** | **4** | **1** | **1** | **1** | **2** | **2** |
| **55** | **3** | **1** | **1** | **1** | **1** | **1** | **0** | **0** | **1** |
| **56** | **3** | **3** | **1** | **2** | **0** | **1** | **0** | **0** | **1** |
| **57** | **3** | **2** | **5** | **3** | **1** | **1** | **0** | **2** | **1** |
| **58** | **3** | **1** | **1** | **1** | **1** | **1** | **0** | **1** | **1** |
| **59** | **2** | **2** | **1** | **4** | **1** | **1** | **0** | **1** | **1** |
| **60** | **3** | **2** | **1** | **5** | **0** | **0** | **0** | **0** | **1** |
| **61** | **3** | **2** | **1** | **2** | **1** | **1** | **0** | **1** | **1** |
| **62** | **3** | **2** | **1** | **6** | **1** | **0** | **1** | **1** | **1** |
| **63** | **3** | **1** | **5** | **2** | **0** | **0** | **0** | **0** | **1** |
| **64** | **3** | **1** | **1** | **1** | **1** | **1** | **0** | **0** | **0** |
| **65** | **3** | **1** | **2** | **2** | **1** | **0** | **0** | **4** | **2** |
| **66** | **3** | **1** | **3** | **1** | **1** | **1** | **0** | **1** | **1** |
| **67** | **2** | **1** | **1** | **3** | **1** | **1** | **0** | **1** | **1** |
| **68** | **2** | **2** | **5** | **4** | **0** | **0** | **2** | **0** | **1** |
| **69** | **3** | **2** | **2** | **7** | **1** | **1** | **0** | **0** | **2** |
| **70** | **3** | **2** | **1** | **1** | **1** | **1** | **2** | **0** | **1** |
| **71** | **3** | **2** | **1** | **6** | **1** | **1** | **0** | **0** | **1** |
| **72** | **3** | **1** | **4** | **2** | **1** | **1** | **0** | **0** | **1** |
| **73** | **3** | **2** | **1** | **1** | **1** | **1** | **2** | **0** | **1** |
| **74** | **3** | **1** | **1** | **1** | **1** | **1** | **2** | **0** | **1** |
| **75** | **3** | **3** | **1** | **5** | **1** | **1** | **2** | **0** | **1** |
| **76** | **3** | **3** | **2** | **7** | **1** | **1** | **2** | **0** | **2** |
| **77** | **3** | **1** | **1** | **2** | **0** | **0** | **2** | **0** | **2** |
| **78** | **3** | **2** | **2** | **1** | **1** | **1** | **0** | **3** | **1** |
| **79** | **3** | **2** | **1** | **4** | **1** | **1** | **0** | **0** | **1** |
| **80** | **3** | **1** | **1** | **1** | **1** | **0** | **2** | **2** | **2** |
| **81** | **2** | **1** | **4** | **1** | **1** | **1** | **2** | **1** | **1** |
| **82** | **2** | **1** | **1** | **2** | **1** | **1** | **2** | **0** | **0** |
| **83** | **2** | **3** | **1** | **2** | **1** | **0** | **2** | **4** | **1** |
| **84** | **2** | **2** | **3** | **4** | **1** | **1** | **2** | **1** | **1** |
| **85** | **2** | **1** | **1** | **4** | **1** | **1** | **2** | **3** | **1** |
| **86** | **3** | **3** | **2** | **6** | **1** | **1** | **0** | **2** | **1** |
| **87** | **3** | **2** | **1** | **5** | **1** | **1** | **2** | **0** | **2** |
| **88** | **3** | **1** | **1** | **2** | **0** | **0** | **2** | **2** | **2** |
| **89** | **3** | **2** | **5** | **1** | **1** | **1** | **0** | **0** | **1** |
| **90** | **3** | **3** | **1** | **2** | **1** | **1** | **2** | **2** | **1** |
| **91** | **2** | **2** | **2** | **2** | **1** | **1** | **2** | **0** | **0** |
| **92** | **2** | **2** | **5** | **1** | **1** | **0** | **2** | **0** | **0** |
| **93** | **2** | **1** | **1** | **4** | **1** | **1** | **0** | **3** | **1** |
| **94** | **3** | **1** | **1** | **1** | **1** | **1** | **2** | **0** | **1** |
| **95** | **3** | **2** | **1** | **6** | **1** | **1** | **1** | **4** | **1** |
| **96** | **3** | **2** | **1** | **3** | **1** | **1** | **0** | **2** | **1** |
| **97** | **3** | **3** | **1** | **2** | **1** | **1** | **1** | **3** | **1** |
| **98** | **3** | **1** | **1** | **2** | **1** | **1** | **2** | **0** | **1** |
| **99** | **2** | **2** | **3** | **6** | **1** | **1** | **1** | **1** | **1** |
| **100** | **3** | **1** | **1** | **7** | **1** | **1** | **1** | **3** | **1** |
| **101** | **3** | **1** | **1** | **3** | **1** | **1** | **1** | **1** | **1** |
| **102** | **2** | **2** | **1** | **1** | **0** | **1** | **2** | **0** | **1** |
| **103** | **2** | **1** | **2** | **2** | **1** | **1** | **0** | **1** | **0** |
| **104** | **3** | **2** | **2** | **5** | **1** | **1** | **2** | **0** | **2** |
| **105** | **3** | **3** | **1** | **2** | **1** | **1** | **1** | **3** | **1** |
| **106** | **3** | **1** | **1** | **7** | **1** | **1** | **0** | **3** | **2** |
| **107** | **3** | **1** | **1** | **5** | **1** | **1** | **2** | **0** | **1** |
| **108** | **3** | **2** | **1** | **2** | **1** | **0** | **2** | **4** | **1** |
| **109** | **3** | **2** | **1** | **5** | **1** | **1** | **2** | **0** | **2** |
| **110** | **3** | **2** | **5** | **2** | **0** | **0** | **0** | **1** | **1** |
| **111** | **2** | **2** | **1** | **1** | **1** | **1** | **1** | **0** | **0** |
| **112** | **3** | **1** | **1** | **5** | **1** | **1** | **2** | **0** | **1** |
| **113** | **3** | **2** | **2** | **6** | **1** | **1** | **0** | **1** | **1** |
| **114** | **3** | **2** | **1** | **1** | **1** | **1** | **2** | **0** | **1** |
| **115** | **2** | **2** | **1** | **5** | **1** | **1** | **2** | **3** | **1** |
| **116** | **3** | **2** | **1** | **6** | **0** | **0** | **0** | **2** | **1** |
| **117** | **3** | **1** | **1** | **2** | **1** | **1** | **0** | **1** | **1** |
| **118** | **3** | **2** | **5** | **1** | **1** | **1** | **2** | **0** | **2** |
| **119** | **2** | **1** | **1** | **6** | **1** | **1** | **1** | **2** | **1** |
| **120** | **2** | **2** | **1** | **4** | **0** | **0** | **0** | **1** | **0** |
| **121** | **2** | **1** | **1** | **2** | **1** | **1** | **0** | **4** | **1** |
| **122** | **2** | **1** | **3** | **2** | **0** | **0** | **2** | **0** | **0** |
| **123** | **2** | **1** | **1** | **6** | **0** | **0** | **1** | **3** | **0** |
| **124** | **2** | **3** | **1** | **3** | **1** | **1** | **2** | **4** | **1** |
| **125** | **2** | **1** | **1** | **3** | **1** | **1** | **0** | **0** | **1** |
| **126** | **2** | **2** | **3** | **1** | **1** | **1** | **2** | **0** | **1** |
| **127** | **2** | **2** | **1** | **1** | **1** | **1** | **1** | **3** | **1** |
| **128** | **2** | **2** | **5** | **1** | **0** | **1** | **2** | **3** | **1** |
| **129** | **2** | **2** | **1** | **4** | **1** | **1** | **2** | **0** | **1** |
| **130** | **3** | **1** | **2** | **1** | **1** | **1** | **0** | **0** | **1** |
| **131** | **2** | **1** | **5** | **1** | **1** | **0** | **2** | **0** | **1** |
| **132** | **2** | **1** | **1** | **2** | **1** | **1** | **0** | **1** | **1** |
| **133** | **3** | **2** | **1** | **4** | **1** | **1** | **2** | **0** | **2** |
| **134** | **3** | **1** | **3** | **1** | **1** | **1** | **2** | **0** | **2** |
| **135** | **3** | **1** | **1** | **3** | **1** | **1** | **2** | **0** | **1** |
| **136** | **2** | **2** | **1** | **5** | **1** | **1** | **0** | **1** | **1** |
| **137** | **2** | **2** | **1** | **6** | **1** | **1** | **0** | **3** | **1** |
| **138** | **3** | **1** | **5** | **3** | **1** | **1** | **2** | **1** | **1** |
| **139** | **2** | **2** | **3** | **1** | **1** | **1** | **0** | **0** | **1** |
| **140** | **1** | **1** | **1** | **2** | **0** | **0** | **0** | **1** | **1** |
| **141** | **3** | **1** | **1** | **7** | **0** | **0** | **0** | **0** | **1** |
| **142** | **2** | **1** | **1** | **1** | **1** | **1** | **0** | **1** | **1** |
| **143** | **3** | **2** | **2** | **3** | **1** | **1** | **0** | **1** | **1** |
| **144** | **2** | **1** | **1** | **1** | **1** | **1** | **0** | **0** | **0** |
| **145** | **3** | **1** | **1** | **1** | **1** | **1** | **0** | **0** | **1** |
| **146** | **2** | **2** | **1** | **1** | **0** | **0** | **1** | **0** | **1** |
| **147** | **3** | **2** | **1** | **3** | **1** | **1** | **0** | **0** | **1** |
| **148** | **3** | **3** | **1** | **6** | **1** | **1** | **1** | **0** | **2** |
| **149** | **2** | **1** | **1** | **4** | **1** | **1** | **0** | **1** | **1** |
| **150** | **3** | **1** | **1** | **2** | **1** | **1** | **0** | **1** | **1** |
| **151** | **3** | **2** | **1** | **1** | **1** | **1** | **0** | **1** | **1** |
| **152** | **2** | **2** | **1** | **1** | **0** | **0** | **0** | **1** | **1** |
| **153** | **3** | **2** | **1** | **4** | **1** | **1** | **1** | **0** | **1** |
| **154** | **2** | **2** | **5** | **1** | **1** | **1** | **0** | **0** | **1** |
| **155** | **1** | **1** | **5** | **2** | **0** | **0** | **0** | **0** | **1** |
| **156** | **3** | **2** | **2** | **2** | **1** | **1** | **0** | **1** | **0** |
| **157** | **2** | **1** | **2** | **1** | **1** | **1** | **0** | **1** | **1** |
| **158** | **3** | **2** | **5** | **1** | **1** | **1** | **0** | **1** | **1** |
| **159** | **3** | **2** | **1** | **1** | **1** | **1** | **0** | **1** | **1** |
| **160** | **3** | **2** | **1** | **1** | **1** | **1** | **0** | **1** | **1** |
| **161** | **2** | **1** | **1** | **3** | **1** | **1** | **0** | **1** | **0** |
| **162** | **2** | **2** | **1** | **4** | **1** | **1** | **0** | **1** | **1** |
| **163** | **2** | **2** | **1** | **2** | **1** | **1** | **1** | **1** | **2** |
| **164** | **3** | **2** | **5** | **1** | **1** | **1** | **0** | **0** | **2** |
| **165** | **3** | **2** | **1** | **1** | **0** | **0** | **0** | **1** | **1** |
| **166** | **3** | **1** | **1** | **1** | **1** | **1** | **0** | **0** | **1** |
| **167** | **2** | **1** | **3** | **1** | **0** | **0** | **1** | **1** | **1** |
| **168** | **3** | **2** | **5** | **2** | **1** | **1** | **0** | **0** | **1** |
| **169** | **2** | **1** | **1** | **1** | **1** | **1** | **0** | **0** | **1** |
| **170** | **2** | **1** | **1** | **1** | **0** | **0** | **0** | **1** | **1** |
| **171** | **2** | **2** | **3** | **4** | **0** | **0** | **0** | **0** | **2** |
| **172** | **3** | **1** | **1** | **2** | **1** | **1** | **0** | **0** | **1** |
| **173** | **2** | **1** | **1** | **2** | **1** | **1** | **0** | **0** | **1** |
| **174** | **3** | **1** | **5** | **1** | **1** | **1** | **0** | **0** | **1** |
| **175** | **2** | **2** | **1** | **6** | **1** | **1** | **0** | **3** | **1** |
| **176** | **3** | **1** | **3** | **1** | **1** | **1** | **0** | **0** | **1** |
| **177** | **3** | **1** | **1** | **2** | **1** | **1** | **0** | **3** | **1** |
| **178** | **3** | **1** | **1** | **1** | **1** | **1** | **0** | **0** | **1** |
| **179** | **2** | **2** | **1** | **1** | **1** | **1** | **0** | **3** | **1** |
| **180** | **2** | **2** | **1** | **3** | **1** | **1** | **0** | **3** | **1** |
| **181** | **3** | **1** | **1** | **1** | **1** | **1** | **0** | **0** | **2** |
| **182** | **3** | **1** | **1** | **2** | **0** | **0** | **0** | **0** | **1** |
| **183** | **2** | **1** | **1** | **2** | **1** | **1** | **0** | **2** | **1** |
| **184** | **3** | **2** | **1** | **3** | **1** | **1** | **0** | **0** | **1** |
| **185** | **2** | **2** | **1** | **2** | **1** | **1** | **1** | **0** | **1** |
| **186** | **3** | **1** | **1** | **1** | **1** | **1** | **0** | **0** | **1** |
| **187** | **3** | **2** | **1** | **2** | **1** | **0** | **1** | **3** | **1** |
| **188** | **3** | **2** | **1** | **2** | **1** | **1** | **0** | **0** | **1** |
| **189** | **1** | **3** | **1** | **6** | **0** | **0** | **0** | **4** | **2** |
| **190** | **2** | **2** | **1** | **2** | **1** | **1** | **1** | **0** | **1** |
| **191** | **3** | **2** | **2** | **3** | **1** | **1** | **0** | **0** | **1** |
| **192** | **3** | **1** | **1** | **1** | **1** | **1** | **0** | **0** | **2** |
| **193** | **3** | **2** | **2** | **1** | **1** | **1** | **0** | **0** | **1** |
| **194** | **3** | **1** | **2** | **2** | **1** | **1** | **0** | **0** | **0** |
| **195** | **2** | **2** | **3** | **6** | **0** | **0** | **1** | **0** | **0** |
| **196** | **2** | **1** | **1** | **4** | **1** | **1** | **0** | **0** | **1** |
| **197** | **3** | **1** | **2** | **4** | **1** | **1** | **0** | **0** | **2** |
| **198** | **2** | **2** | **2** | **6** | **1** | **1** | **0** | **4** | **2** |
| **199** | **3** | **2** | **1** | **2** | **1** | **1** | **0** | **3** | **1** |
| **200** | **3** | **2** | **2** | **2** | **1** | **1** | **0** | **0** | **0** |
| **201** | **3** | **3** | **2** | **2** | **1** | **1** | **2** | **2** | **1** |
| **202** | **3** | **1** | **1** | **2** | **1** | **0** | **0** | **0** | **2** |
| **203** | **2** | **1** | **3** | **1** | **0** | **0** | **2** | **0** | **0** |
| **204** | **3** | **1** | **1** | **1** | **1** | **1** | **0** | **0** | **1** |
| **205** | **2** | **1** | **1** | **1** | **1** | **1** | **0** | **0** | **1** |
| **206** | **3** | **2** | **1** | **1** | **1** | **1** | **0** | **0** | **1** |
| **207** | **3** | **2** | **1** | **1** | **1** | **1** | **0** | **0** | **1** |
| **208** | **2** | **2** | **1** | **1** | **1** | **0** | **0** | **0** | **1** |
| **209** | **1** | **2** | **1** | **6** | **0** | **0** | **0** | **0** | **0** |
| **210** | **3** | **2** | **1** | **1** | **1** | **0** | **1** | **0** | **1** |
| **211** | **3** | **1** | **1** | **3** | **1** | **1** | **0** | **0** | **1** |
| **212** | **3** | **1** | **1** | **1** | **0** | **0** | **0** | **0** | **1** |
| **213** | **3** | **1** | **1** | **7** | **1** | **1** | **0** | **0** | **1** |
| **214** | **3** | **1** | **1** | **2** | **1** | **1** | **0** | **0** | **1** |
| **215** | **2** | **1** | **1** | **1** | **1** | **1** | **0** | **0** | **1** |
| **216** | **3** | **2** | **2** | **3** | **1** | **1** | **0** | **0** | **1** |
| **217** | **2** | **1** | **1** | **2** | **1** | **1** | **0** | **0** | **0** |
| **218** | **3** | **2** | **1** | **1** | **1** | **1** | **1** | **0** | **1** |
| **219** | **3** | **1** | **1** | **1** | **1** | **0** | **0** | **0** | **1** |
| **220** | **3** | **1** | **1** | **1** | **1** | **1** | **0** | **0** | **1** |
| **221** | **2** | **1** | **1** | **7** | **1** | **1** | **0** | **0** | **1** |

| **Clinical Parameters** |  |  |
| --- | --- | --- |
|  |  | **Abbreviations** |
| **Age:** | 20 - 40 years | 1 |
|  | 41 - 60 years | 2 |
|  | 61 - >80 years | 3 |
| **Diagnosis Mamma-Ca:** | right breast | 1 |
|  | left breast | 2 |
|  | both | 3 |
| **Histology:** | duktal | 1 |
|  | lobulär | 2 |
|  | DCIS | 3 |
|  | LCIS | 4 |
|  | mixed and other tumour types | 5 |
| **TNM:** | stadium I | 1 |
|  | stadium IIa | 2 |
|  | stadium IIb | 3 |
|  | stadium IIIa | 4 |
|  | stadium IIIb | 5 |
|  | stadium IIIc | 6 |
|  | stadium IV | 7 |
| **ER status:** | positive | 1 |
|  | negative | 0 |
| **PR status:** | positive | 1 |
|  | negative | 0 |
| **Her2 status:** | positive | 1 |
|  | negative | 0 |
|  | not define | 2 |
| **Response of chemotherapy:** | no chemotherapy / no data | 0 |
|  | complete remission | 1 |
|  | recidive | 2 |
|  | stable disease | 3 |
|  | Progress | 4 |
| **Survival:** | not detect | 0 |
|  | alive | 1 |
|  | death | 2 |

**Supplementary material:**

Tab. 1: Data of breast cancer patients.

Tab. 2: Explanation of the clinical parameters. The age of the patients is defined as age at the time of recruitment.
